# Prediction of compressional sonic log in the western (Tano) sedimentary basin of Ghana, West Africa using supervised machine learning algorithms

**DOI:** 10.1016/j.heliyon.2023.e20242

**Published:** 2023-09-17

**Authors:** Callistus Nero, Akwasi Acheampong Aning, Sylvester Kojo Danuor, Victor Mensah

**Affiliations:** a Department of Physics, Kwame Nkrumah University of Science and Technology, Ghana; b EVChucks Services Ghana, Ghana

**Keywords:** Support vector machine, Random forest, XGBoost, Sonic log prediction, Machine learning applications, Ensemble and non-ensemble algorithms comparison

## Abstract

Sonic logs are essential for determining important reservoir properties such as porosity, permeability, lithology, and elastic properties, among others, and yet may be missing in some well logging suites due to high acquisition costs, borehole washout, tool damage, poor tool calibration, or faulty logging instruments. This study aims at predicting the compressional sonic log from commonly acquired logs (gamma ray, resistivity, density, and neutron-porosity) in the Tano basin of Ghana using Support Vector Machines (SVM), Random Forest (RF), and Extreme Gradient Boosting (XGBoost) Machine Learning (ML) algorithms and comparing the performances of the algorithms. The algorithms were trained with 70% of the data from two wells and tested using the remaining 30% of the data from the wells after cross-validation. Subsequently, they were applied to the data from a third well to predict the sonic log in the well. The performances of the algorithms were assessed with five statistical tools: coefficient of determination (R^2^), adjusted R^2^, Mean Squared Error (MSE), Mean Absolute Error (MAE), and Root Mean Squared Error (RMSE). All three algorithms successfully predicted the compressional sonic log (DT). XGBoost demonstrated the highest prediction accuracy, with R^2^ of 0.9068 and the least errors. RF exhibited the next highest accuracy, with R^2^ being 0.85478, while SVM had R^2^ of 0.66591. Therefore, the ensemble algorithms (XGBoost and RF) proved to be more accurate than the non-ensemble algorithm (SVM) in this study. The outcome of the study will accelerate and enhance the understanding of oil and gas fields with few or no compressional sonic logs. To the best of the authors’ knowledge, this is the first study to have predicted the compressional sonic log from well data (logs) in a Ghanaian sedimentary basin using machine learning algorithms, and only a few such studies have been conducted in the whole West African sub-region.

## 1 Introduction

Sonic logs are essential for determining important reservoir properties such as porosity, permeability, lithology, and elastic properties, among others, and yet may be missing in some well logging suites due to high acquisition costs, borehole washout, tool damage, poor tool calibration, or faulty logging instruments [1,2]. These properties affect the accumulation and migration of petroleum in a reservoir. The elastic properties are particularly useful for well placement and reservoir productivity optimization [3]. The absence of this log in a reservoir may therefore lead to a poor understanding of the reservoir and, consequently, poor reservoir evaluation and performance.

Despite this risk, the high cost of acquisition of sonic logs may deter companies from acquiring them in future oil and gas projects owing to the increasing difficulty with obtaining funding for hydrocarbon projects resulting from the global energy transition (the global shift from fossil-based fuels to non-carbon energy sources).

In cases where the sonic log was missing in a logging suite, it was obtained by transforming the commonly acquired logs, such as resistivity and density logs, based on an experimental formula built between them. However, this approach is prone to unacceptable errors as the formula may not be suitable for all formation conditions across oil and gas fields [1]. This necessitated the use of Machine Learning (ML) techniques to study reservoirs and synthesize well logs since these techniques are capable of modeling seemingly unrelated parameters and solving nonlinear problems.

ML is a subdivision of Artificial Intelligence (AI) that uses various algorithms to teach computers to identify patterns in data and based on the patterns, make predictions and forecasts. These algorithms mimic human cognitive functions such as learning, reasoning, problem-solving, and self-correction and serve as a quality check for performance optimization [4]. ML algorithms may be time- and cost-saving, faster, more accurate, and cheaper than traditional reservoir characterization technologies. Therefore, relying on them for oil and gas exploration and production has the potential of lowering exploration risk, minimizing cost, and maximizing oil and gas production. Speed and precision are indispensable in the global oil and gas industry under the prevailing conditions of energy transition, which is why ML ought to be given the utmost attention in the industry.

Several studies attest to the ability of ML algorithms to synthesize or predict sonic logs based on commonly acquired logs such as gamma ray, density, neutron-porosity, resistivity, and spontaneous potential, among others [5]. relied on supervised ML algorithms to predict shear sonic log based on gamma ray, neutron-porosity, bulk density, true resistivity, compressional sonic, computed total porosity, and water saturation logs from two vertical wells located in the Poseidon field in the Browse Basin, offshore North West Australia. In a different study [2], recognizing that sonic logs may be missing in some well logging suites, used Support Vector Regression (SVR) to predict them in wells in the Anadarko Basin, Oklahoma, based on deep resistivity, gamma ray, density, self-potential and photoelectric logs. They advised that models with multiple variables should be used for sonic logs and other predictions because they are more reliable than models with single variables. In another study in the Anadarko Basin (Oklahoma) [6], relied on gamma ray and deep resistivity logs and the Gene Expression Programming algorithm to predict sonic logs. In a related study [1], used Kernel Extreme Learning Machine to predict sonic logs in seven wells in a GJH survey in the Erdos Basin. They used gamma ray, deep resistivity, density, and spontaneous potential logs as predictors [7]. applied random forest regression methods, support vector regression, and K-nearest neighbours algorithms on density, gamma ray, and porosity logs data obtained from the Netherlands F3 block to predict sonic logs [3]. used Artificial Neural Networks with gamma ray, neutron porosity, and density logs from two wells as input parameters to predict sonic logs.

Apart from these, several other studies have demonstrated the efficacy of ML algorithms in the prediction of other reservoir properties such as porosity [[8], [9], [10]], permeability [[10], [11], [12], [13], [14], [15], [16], [17], [18], [19]], lithologies [[9], [10], [20], [21], [22], [23], [24], [25], [26], [27], [28], [29], [30]], sand volumes [31], wave velocities [[32], [33], [34]] and formation bulk density [35] among others. All the above studies and many others employed ML algorithms such as SVM, Random Forest, XGBoost, AGBoost, Artificial Neural Networks (ANN), etc., to predict various reservoir properties.

Similar to the aforementioned studies, this study aims at predicting the compressional sonic log by applying SVM and two ensemble machine learning algorithms (random forest and XGBoost) on commonly acquired logs (gamma ray, density, neutron-porosity, and resistivity) from three wells in the Western (Tano) Sedimentary Basin of Ghana taking cognizance of the relevance of the sonic log in oil and gas exploration and production, its high cost of acquisition and the threats imposed by the global energy transition. The study also aims at establishing the veracity of claims that the ensemble machine learning algorithms are more accurate than the non-ensemble algorithms [36,37] by comparing the performance of the SVM algorithm (a non-ensemble algorithm) with the ensemble algorithms used. The prediction of the compressional sonic log will be carried out by training and testing the algorithms using data from two wells and subsequently further validating their accuracies by deploying them on a blind data set (data from a third well). The performances of the algorithms will be assessed using five statistical tools.

This study is imperative to reassure hydrocarbon exploration and production companies, as well as nascent oil and gas nations, that hydrocarbon exploration and production can be achieved with limited or no sonic log information. The outcome of the study will accelerate and enhance the understanding of oil and gas fields with few or no compressional sonic logs, as this study will motivate the generation of synthetic sonic logs or any other logs that may be missing in a logging suite. Predicting logs instead of acquiring them also saves time and cost, which could lead to optimized oil and gas exploration and production and potentially lower related carbon dioxide emissions as envisaged by the global energy transition agenda.

To the best of the knowledge of the authors, this is the first time ML algorithms are employed to predict the compressional sonic log in a Ghanaian sedimentary basin. The outcome of this study will, therefore, be useful for the ongoing exploration works and the unexplored portions of the Western Basin as well as other sedimentary basins in Ghana. The results could help accelerate oil and gas exploration and production in Ghana to generate the necessary revenues to support the development of the clean energy resources (little or no carbon energy sources) envisaged by the energy transition.

## 2 Overview of the ML algorithms used in this study

### 2.1 Support vector machines

SVMs are supervised machine learning models with a kernel function for non-linear classification and regression. With this algorithm, linear learning machines are trained in kernel-induced feature spaces by applying the generalization theory of Vapnik-Chervonenkis [38,39]. SVMs have high efficiency and good generalization performance. They also have strong adaptability, rigorous theory, and global optimization [31]. In the case of classification, they find a hyperplane in N-dimensional space that distinctly classifies the data points [40]. According to Ref. [41], predictions with SVM are based on the functional form equation:

(1) $$y\left(x\right)=\sum_{n=1}^{N}w_{n}K\left(x,x_{n}\right)+w_{0}$$

Where $w_{n}$ and w_0_ represent the model ‘weights’ and $K\left(x,x_{n}\right)$ represents the kernel function.

According to Ref. [2], the SVR methodology has strong nonlinear approximation capabilities, good generalization effectiveness, and does not overpredict mean values, and preserves original data variability as compared to traditional deterministic methods. The SVR also deals greatly with data uncertainty, big data size, and data diversity.

### 2.2 Random forest

The RF algorithm builds an ensemble of trees (many independent decision trees), trains them, and subsequently carries out predictions with them. It prevents overfitting using bootstrap re-sampling. Bootstrap sets are built on initial data with several samples replacing other repeating samples. An individual bootstrap set produces a tree (e.g., N different bootstrap representations are needed for N tree estimators). The trees built, therefore, have different datasets and predictions. For regression, the different trees are aggregated after training, and the predictions of each tree are averaged to obtain the final predictions [42,43]. According to Ref. [31], the RF algorithm is accurate, tolerant to outliers and noise, and does not easily overfit.

To predict at a point x:

(2) $$\text{Regression}:\hat{⨍}\begin{array}{l} B\\ rf \end{array}\left(x\right)=\frac{1}{B}\sum_{b=1}^{B}T_{b\left(x\right)}$$

Classification: ${Ĉ}_{rf}^{B}\left(x\right)=$ majority vote ${\left\{{Ĉ}_{b}\left(x\right)\right\}}_{1}^{B}$ , where ${Ĉ}_{b}\left(x\right)$ = prediction of the bth random-forest tree.

Where $T_{b}$ is a random forest tree, B is the number of trees.

### 2.3 Extreme gradient boost

XGBoost is a combination of tree ensemble learning and gradient descent. This supervised learning model can be used for regression and classification problems. The performance of the XGBoost model is improved by adding one new tree at a time to the previous XGBoost model. This also optimizes the value of a pre-specified objective function [31]. It avoids overfitting by making use of the regularization term:

(3) $$y_{i}^{\left(t\right)}=\sum_{k=1}^{t}f_{k}\left(x_{i}\right)=y_{i}^{\left(t-1\right)}+f_{t}\left(x_{i}\right)$$

*t* = total number of base tree models, $y_{i}^{\left(t\right)}$ = final tree model, $y_{i}^{\left(t-1\right)}$ = previously generated tree model, and $f_{t}\left(x_{i}\right)$ = newly generated tree model.

The random forest and XGBoost algorithms were randomly selected from the ensemble family of ML algorithms based on the assertion that the ensemble methods are generally robust. However, the two algorithms employ different techniques for making predictions. While random forest uses bagging or bootstrap aggregation, XGBoost uses boosting. In bagging, many independent models are built, and the average value is used while boosting builds models sequentially [4]. Both approaches reduce bias and variance.

The regression versions of all three algorithms were used to predict the compressional sonic log in this study.

## 3 Study area

**Fig. 1:**
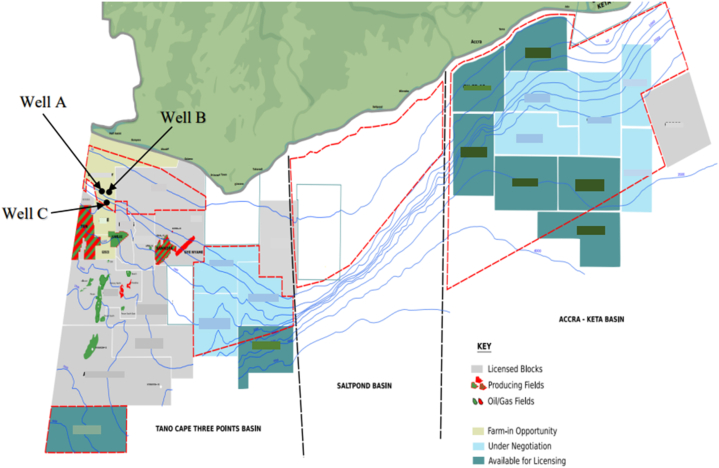
Map of Ghana Offshore Basins showing study area (modified from Petroleum Commission).

The wells used for this study are located in the Western (Tano) Cape Three Points Basin of Ghana (West Africa) (Fig. 1). The Western Basin is a cretaceous wrench-modified pull-apart basin formed as a result of the *trans*-tensional movement during the separation of Africa and South America and the opening of the Atlantic in the Albian. Active rifting and subsidence during this period resulted in the formation of a deep basin characterized by thick organic-rich shale in the Cenomanian and Turonian. Large turbidite and channel complexes were deposited as a result of several river systems [44]. The Western Basin is located in the western part of Ghana and is the country's most prolific sedimentary basin. It is home to Ghana's three producing fields and several oil and gas discoveries. This notwithstanding, several acreages within the basin are still under exploration, and deeper portions of it remain unexplored.

Therefore, the study's outcome will be useful for the ongoing exploration works and the unexplored portions of the basin as well as the other sedimentary basins of the country with little or no well data.

## 4 Methodology

**Fig. 2:**
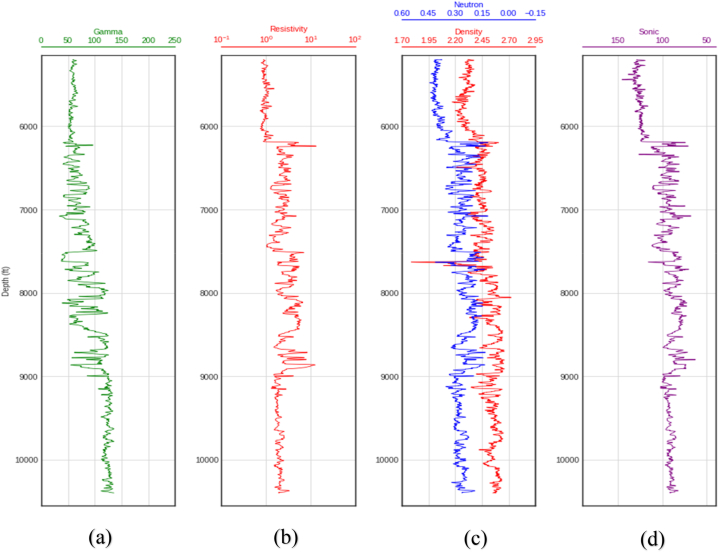
Wireline log plots for well A. (a) Gamma ray log (b) Resistivity log (c) Neutron-porosity/Density logs (d) Sonic log.

**Fig. 3:**
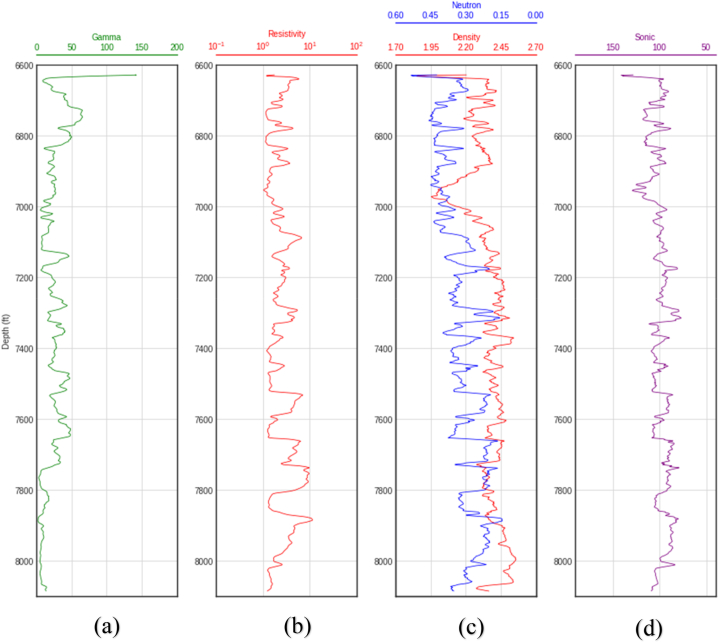
Wireline log plots for well B. (a) Gamma ray log (b) Resistivity log (c) Neutron-porosity/Density logs (d) Sonic log.

**Fig. 4:**
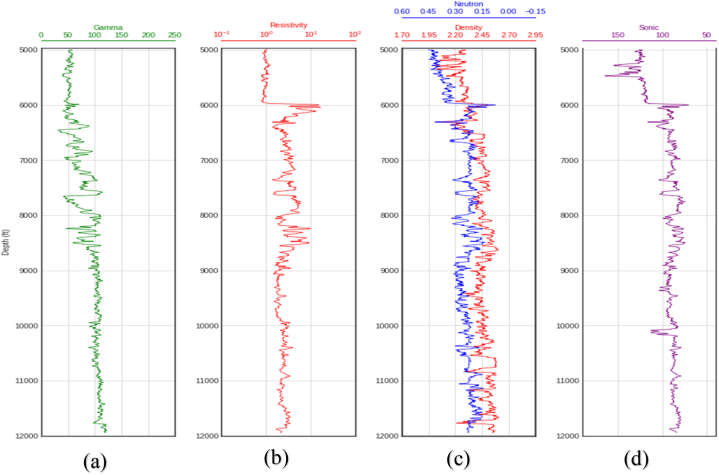
Wireline log plots for well C. (a) Gamma ray log (b) Resistivity log (c) Neutron-porosity/Density logs (d) Sonic log.

The wells used for this study are labelled A, B and C and contained Gamma Ray (GR), Neutron-Porosity (NPHI), Density (RHOB), Resistivity (RES), and Sonic (DT) logs each. The log plots for the three wells are shown in Fig. 2, Fig. 3, Fig. 4, and Table 1 is a statistical summary of the data.

**Table 1:** Statistical summary of the logs.

	DEPTH	GR	RES	RHOB	NPHI	DT
COUNT	20274	20274	20274	20274	20274	20274
MEAN	7963.112544	77.992301	2.559616	2.440367	0.277498	96.555557
STD	1649.021858	34.294738	1.630676	0.119845	0.071758	14.594731
MIN	4998.999000	2.057459	0.736288	1.790253	0.075000	62.812741
25%	6739.624000	53.632597	1.629784	2.367937	0.225000	87.082663
50%	7754.249000	76.092379	2.162194	2.451535	0.265409	92.324459
75%	9223.999000	108.102034	2.984840	2.531176	0.317701	101.681937
MAX	11941.999000	142.079950	16.722424	2.722547	0.533925	164.352929

From Table 1, GR values range between 2 and 142 API, indicating varying lithologies within the formation. RES values range between 0.74 and 16.72 Ωm, depicting the composition of the formation and the different fluids that might be contained within the formation. The values of RHOB indicate the presence of different subsurface layers with densities varying between 1.79 and 2.7 g/cm^3^, while the NPHI values ranging between 0.07 and 0.53 indicate the presence of both low and high porosities at different locations within the reservoir. DT values range between 62.8 and 164.4 μs/ft, signifying different layer velocities and the nature of the sediments within the layers.

**Fig. 5:**
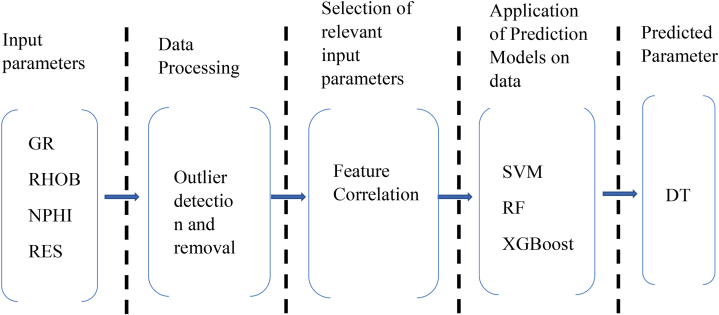
Compressional sonic log or travel times prediction workflow.

Prior to the deployment of the ML algorithms on the data to predict DT, the data were preprocessed to identify and remove outliers. Subsequently, the data underwent a feature correlation process to reduce redundancy and dimensionality of the input parameters to enhance the performance of the models. Fig. 5 provides the workflow used in this study to predict DT.

### 4.1 Outlier detection and removal

Logs or well data may contain unwanted data points or noise as a result of several factors, including human errors and poor instrument calibrations. Consequently, the data from the three wells were processed to identify and remove outliers using histograms and the isolation forest algorithm.

**Fig. 6:**
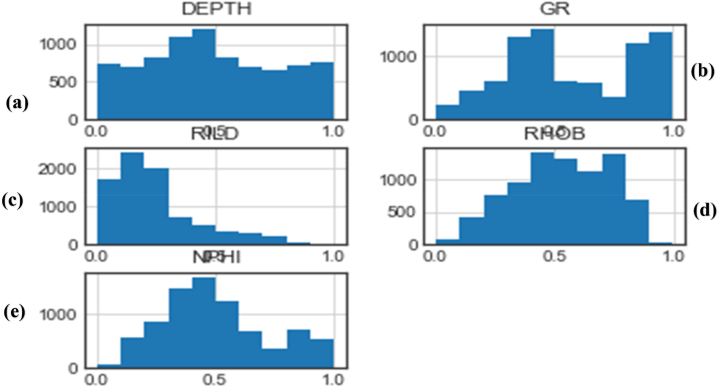
Histograms of the different training input parameters showing the data distribution. (a) Depth distribution (b) Gamma Ray log distribution (c) Resistivity log distribution (d) Density log distribution (e) Neutron-porosity log distribution.

Histograms provide information on the distribution of the various variables and enable visual identification of the outliers [45]. Fig. 6 below gives the distribution of the logs used for this study.

**Fig. 7:**
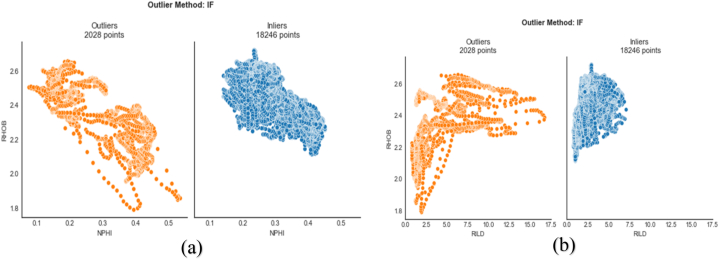
Outlier detection using isolation forest. The orange spots are the outliers. (a) Outliers and inliers of neutron-porosity log (b) Outliers and inliers of resistivity log.

The isolation forest algorithm uses isolation (how dispersed the data points are) to detect anomalies. It requires a low memory and has a linear time complexity with a low constant. For a given data set, Isolation Forest builds an ensemble of iTrees and regards instances with short average path lengths on the iTrees as anomalies [26]]. Fig. 7 shows the outliers and inliers based on isolation forest detection. Out of a total of 20274 data points (Table 1), 18246 data points were valid (inliers), while 2028 data points were outliers. For each parameter (the well logs), data points below the minimum value and above the maximum value in Table 1 are considered outliers.

### 4.2 Feature correlation

**Fig. 8:**
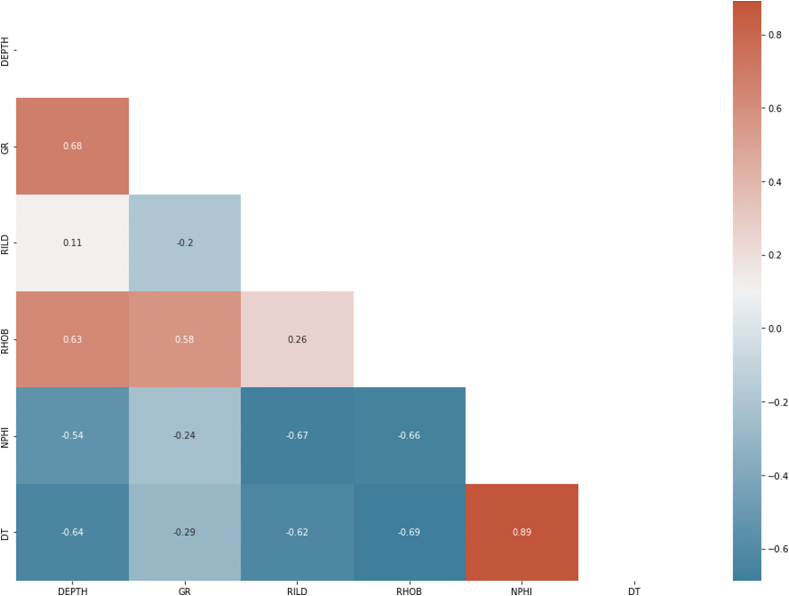
Pearson heat map correlation.

The aim of feature correlation is to identify and remove irrelevant and redundant input parameters to improve the performance of the algorithms. This was achieved using the Pearson Correlation Heat Map to establish collinear relationships between the input parameters. According to Ref. [4], for any two input parameters with ±90% collinearity, one of the parameters will be dropped because such parameters provide the same information and hence would result in redundancy and increased dimensionality of the input data set if they are both kept. From the heat map (Fig. 8), the correlation coefficient between any two input parameters is below the ±90% benchmark, indicating that all the input parameters will provide the algorithms with unique information and, therefore, deemed relevant for the study. Based on this observation, all the parameters (well logs) were used as inputs in this study.

The data were also normalized or standardized to improve their integrity and prevent the algorithms from being biased to the magnitude of the data. The choice of a normalization method depends on the application and the algorithm [46]. According to Ref. [4], algorithms such as SVM and neural networks require normalized data to function correctly. Consequently, the data used for this study were normalized using the min-max scaler because of the SVM algorithm. This involves scaling features or the data to lie between a given minimum and maximum value. The normalization was in the range of 0–1 according to the following equation:

(4) $$x_{norm}=\frac{x-x_{\min}}{x_{max}-x_{\min}}$$

Where $x_{norm}$, $x_{\max}$ and $x_{\min}$ are the normalized, maximum, and minimum input data respectively.

Following these processes, the SVM, RF and XGBoost algorithms were trained and tested using the data from wells A and B. Training and testing or validation of ML algorithms are essential to provide assurance that they can produce similar or the desired results when applied to other data sets in different locations. There are several ways of validating ML algorithms, and this study adopted the holdout or random sampling and blind set validation methods after some preliminary investigations. The holdout or random sampling method involves randomly splitting the data set into two portions: one portion for training the algorithms and the other for testing them. In this study, seventy percent (70%) of the data from wells A and B were randomly selected and used to train the algorithms and the remaining thirty percent (30%) were used to test them.

To further assess the credibility of the results produced by the holdout method, the K-fold cross-validation was also implemented on the data from wells A and B, and the results were compared with those of the holdout method. The K-fold cross-validation is an elaboration of the holdout method which divides the data into K-subsets or folds and, then the holdout method is repeated K-times. In this case, the data from wells A and B were divided into ten folds or subsets, following which ten rounds of predictions were carried out. At each round, one fold or subset was used to test the algorithms after the remaining nine folds were used to train them, and the accuracy was determined by averaging the results of the ten predictions.

The algorithms were further validated by implementing the blind set validation. This involves applying the algorithms to a data set that has not been used for training or testing to ensure an unbiased evaluation of the algorithms’ accuracies. In this study, the data from well C constituted the blind data set to which the SVM, RF, and XGBoost algorithms were applied after the holdout and K-fold methods were carried out or after the training and testing were conducted.

### 4.3 Setting hyperparameters for the algorithms

Machine learning algorithms have parameters that have to be optimized to ensure accurate predictions and avoid overfitting or underfitting. These parameters are adjusted during training and testing until an algorithm produces the best performance or the most accurate predictions. The parameters that produce the best model's performance are called hyperparameters and are used to configure the algorithms and control the learning process. They produce the minimum generalization error on the test set. The model overfits if it is too complex and underfits if it is too simple (it does not capture most of the complexity of the data). Under these circumstances, the generalization error will be very high [31].

In this study, the hyperparameters of the SVM, RF, and XGBoost algorithms were determined using the grid search method. This involves searching through a wide variety of hyperparameter combinations to find the combination that returns the highest model performance. The parameters that returned the models with the highest accuracies in this study are contained in Table 2 below and were used for the various predictions.

**Table 2:** Optimal hyperparameters of the algorithms.

Algorithm	Hyperparameters
SVM	Kernel = rbf gamma = 1.5C = 5
XGBoost	obj = reg:squared error, n-estimators = 200, reg-lambda = 1, gamma = 0, max-depth = 3, learning rate = 0.1, reg-alpha = 0.1
RF	n-estimators = 500, criterion = mse, max-depth = none, min-samples-split = 4, min-samples-leaf = 2, max-features = 1, boostrap = true, n-jobs = −1

The performances of the algorithms were assessed based on the correlation between the predicted sonic logs and the original sonic logs in the wells using five statistical tools: coefficient of determination (R^2^), adjusted R^2^ ($R_{adj}^{2}$), Mean Squared Error (MSE), Mean Absolute Error (MAE) and Root Mean Squared Error (RMSE). MAE depicts the average of the absolute value of the difference between the actual and predicted values. MSE is the mean of the squared errors and RMSE depicts the square root of the mean squared errors. These tools indicate how well the predicted sonic log correlates with the actual or measured sonic log.

(5) $$MAE=\frac{1}{n}\sum_{i=1}^{n}{|x}_{i}-{\hat{y}}_{i}|$$

(6) $$MSE=\frac{1}{n}\sum_{i=1}^{n}{\left(x_{i}-{\hat{y}}_{i}\right)}^{2}$$

(7) $$RMSE=\sqrt{\frac{1}{n}}\sum_{i=1}^{n}{\left(x_{i}-{\hat{y}}_{i}\right)}^{2}$$

Where n is the number of sample points, $x_{i}$ is the ith sample point output and ${\hat{y}}_{i}$ is the predicted value.

(8) $$R=\frac{\sum \left(x_{i}-\bar{x}\right)\left(y_{i}-\bar{y}\right)}{\sqrt{\sum {\left(x_{i}-\bar{x}\right)}^{2}\sum {\left(y_{i}-\bar{y}\right)}^{2}}}$$

$x_{i}$ - Measured value at point *i*, $\bar{x}$ – mean of the measured values [47]

$y_{i}$ – Predicted value at point *i*, $\bar{y}$ – mean of the predicted values

(9) $$R^{2}={\left(\frac{\sum \left(x_{i}-\bar{x}\right)\left(y_{i}-\bar{y}\right)}{\sqrt{\sum {\left(x_{i}-\bar{x}\right)}^{2}\sum {\left(y_{i}-\bar{y}\right)}^{2}}}\right)}^{2}$$

(10) $$R_{adj}^{2}=1-\frac{\left(1-R^{2}\right)\left(n-1\right)}{n-p-1}$$

where p is the number of predictors, and n is the number of sample points[16].

## 5 Results and discussions

The results of the holdout and K-fold cross-validation methods during training and testing of the algorithms with the data from wells A and B are presented in Table 3 below. The holdout method (R^2^ of 0.94784) slightly outperformed the K-fold (R^2^ of 0.94781) during training of SVM but both methods had the same accuracy (R^2^ of 0.94783) during testing. As opposed to the SVM, K-fold slightly outperformed the holdout method in both training and testing of RF. The difference was about 0.01% for the training and 0.001% for the testing. The accuracies were the same for both cross-validation methods when XGBoost was trained and tested.

**Table 3:** Performance comparison of the holdout and K-fold cross-validation methods.

Algorithm	Training		Testing	
	Holdout (R^2^)	K-fold (R^2^)	Holdout (R^2^)	K-fold (R^2^)
SVM	0.94784	0.94781	0.94783	0.94783
RF	0.9979	0.9980	0.99404	0.99405
XGBoost	0.99013	0.99013	0.9890	0.9890

Given the similarity in the results of both cross-validation methods and the high accuracies recoded, the algorithms were considered capable of predicting the sonic log from a blind data set.

**Fig. 9:**
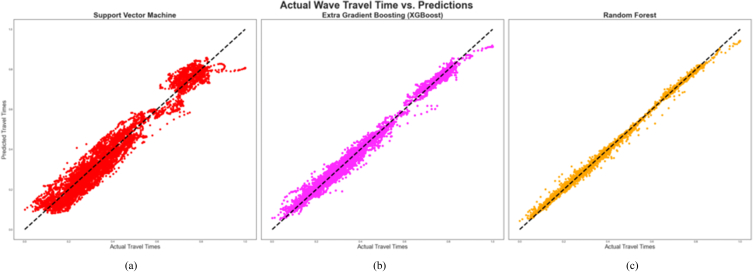
Cross plot of predicted and actual compressional wave travel times during training of the algorithms. (a) Training of SVM (b) Training of XGBoost (c) Training of Random Forest.

**Fig. 10:**
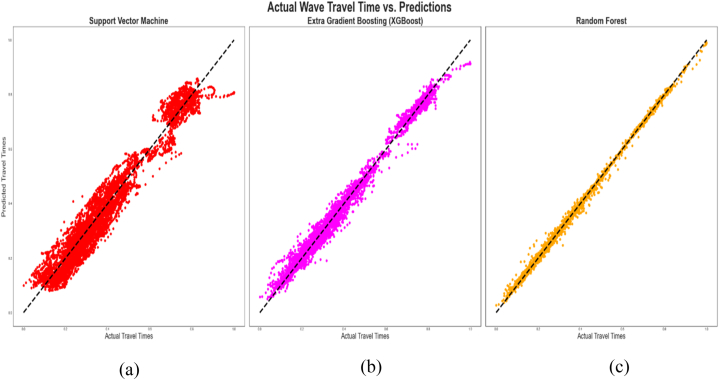
Cross plot of predicted and actual compressional wave travel times during testing of the algorithms. (a) Testing of SVM (b) Testing of XGBoost (c) Testing of Random Forest.

However, only the results of the holdout cross-validation for training and testing the algorithms have been provided in this study because it outperformed the K-fold when the SVM was used for the predictions considering that the SVM operates on a different principle from XGBoost and RF. These results are Table 4, Table 5 and Fig. 9, Fig. 10 below. Table 4, Table 5 contain the statistical tools that depict the degree of correlation or deviation between the actual or measured and the predicted data points during the training and testing of the algorithms respectively. Table 6 also contains the statistical tools that were used to assess the performance of the algorithms during the blind set validation of the algorithms.

**Table 4:** Performance comparison of the different algorithms (Training of algorithms).

Algorithm	R^2^	$R_{adj}^{2}$	MAE	MSE	RMSE
SVM	0.9478	0.9478	0.03560	0.00196	0.04425
XGBoost	0.9901	0.9901	0.01362	0.00037	0.01925
RF	0.9979	0.9979	0.00318	0.00004	0.00612

**Table 5:** Performance comparison of the different algorithms (Testing of algorithms).

Algorithm	R^2^	$R_{adj}^{2}$	MAE	MSE	RMSE
SVM	0.9478	0.9478	0.03562	0.00193	0.04393
XGBoost	0.9890	0.9889	0.01446	0.00041	0.02017
RF	0.9940	0.9940	0.00685	0.00016	0.01260

**Table 6:** Performance comparison of the different algorithms (validation of algorithms).

Algorithm	R^2^	$R_{adj}^{2}$	MAE	MSE	RMSE
SVM	0.66591	0.6656	0.07562	0.01	0.09998
XGBoost	0.90688	0.90680	0.03590	0.00279	0.05278
RF	0.85478	0.85476	0.04059	0.00434	0.06591

**Fig. 11:**
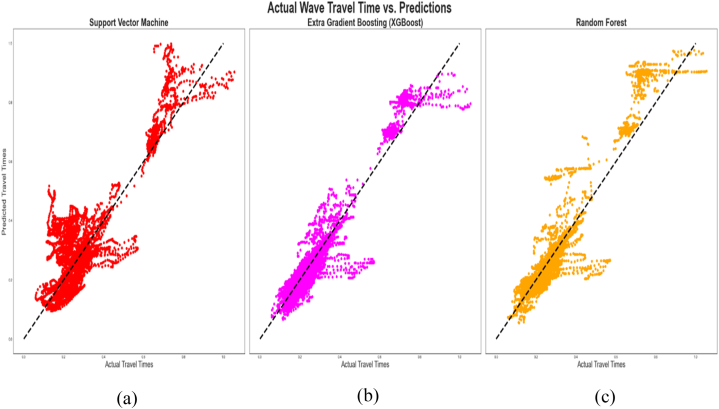
Cross plot of predicted and actual compressional wave travel times on blind data (validation). (a) Validation of SVM (b) Validation of XGBoost (c) Validation of Random Forest.

**Fig. 12:**
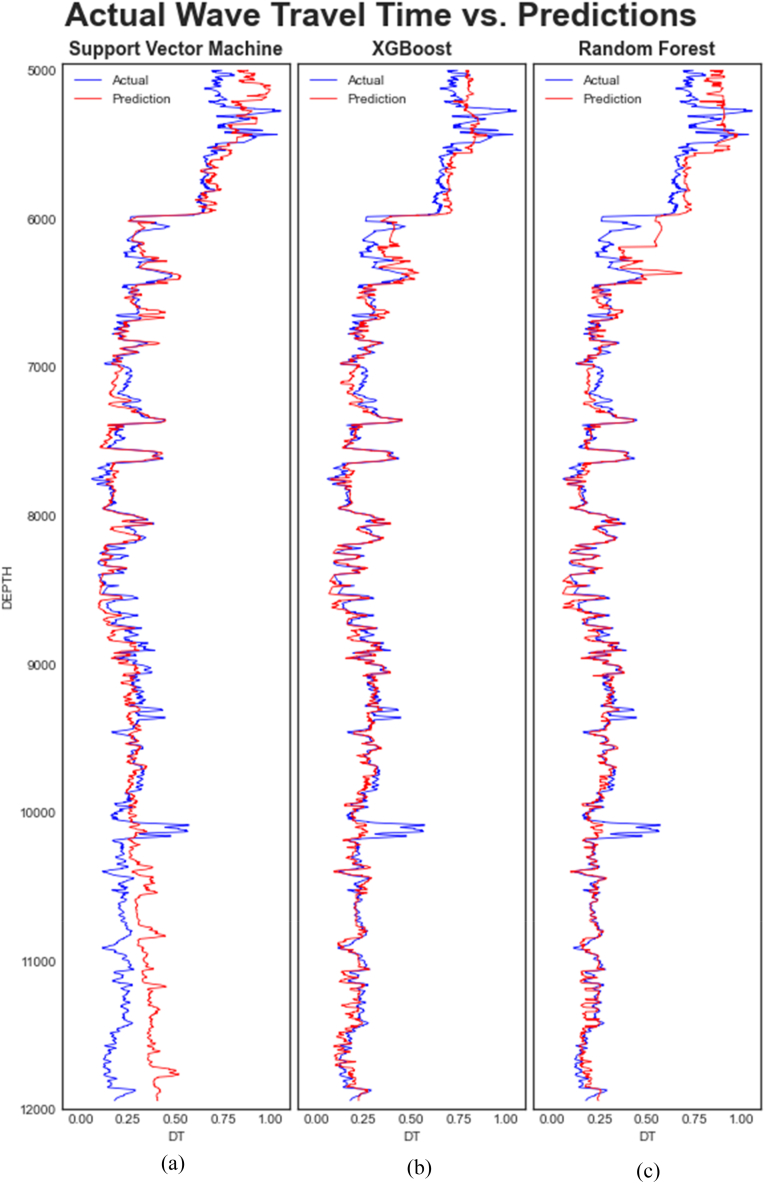
Comparison of measured (actual) and predicted Compressional wave travel times on blind data (well C). (a) Validation of SVM (b) Validation of XGBoost (c) Validation of Random Forest.

Fig. 9, Fig. 10 are cross plots of the actual and predicted travel times (compressional sonic log) during training and testing of the algorithms respectively while Fig. 11 is the cross plot of the predicted and actual travel times during the blind set validation. In all three figures, the actual travel times are represented by the X-axis, while the predicted travel times are represented by the Y-axis. Fig. 12 is a plot of the predicted and actual compressional wave sonic logs in well C when the blind set validation was carried out.

From Fig. 9, Fig. 10, a strong correlation is observed between the actual and predicted data points, as evidenced by the clustering of the data points diagonally or along the mean line. This means that the predictions carried out by the algorithms were highly accurate during training and testing. This observation is supported by Table 4, Table 5, which provide the coefficients of determination and the adjusted coefficients of determination between the actual and predicted travel times, as well as the associated errors of the predictions during training and testing of the algorithms. From these tables, the RF algorithm exhibited the highest accuracy, having the highest R^2^ of 0.9979 and 0.9940 during training and testing respectively. This means that 99.79% of the predicted data correlated with the actual data during training, and 99.40% of the predicted data correlated with actual data when RF was applied to the remaining 30% of the data from wells A and B (testing). The R^2^ values were validated by calculating the adjusted R^2^ $\left(R_{adj}^{2}\right)$ values, which were very similar to the R^2^ values for both training and testing. Correspondingly, the RF algorithm recorded the least errors, as evidenced by the MAE, MSE, and RMSE values in Table 4, Table 5 The XGBoost algorithm exhibited the second-highest accuracy, having 99.01% of its predicted data correlating with the actual data during training and 98.9% of its predicted data correlating with the actual data points when it was tested on the remaining 30% of the data from wells A and B. It also recorded lower MAE, MSE, and RMSE values than the SVM algorithm. In the case of SVM, 94.78% of the predicted data points correlated with the actual data points during both training and testing (Table 4, Table 5). It recorded higher errors than XGBoost and RF during training and testing, making it exhibit the lowest accuracy, albeit above 90%.

In Fig. 11 which displays the predicted and actual travel times when the algorithms were applied on the blind data set (well C), majority of the data points have also clustered along the mean line but the data points are more scattered as compared to the performances of the algorithms during training and testing (Fig. 9, Fig. 10). This observation aligns with the relatively lower prediction accuracies of the algorithms presented in Table 6 below. From Table 6, XGBoost demonstrated the best performance, having R^2^ and $R_{adj}^{2}$ of 0.90688 and 0.90680 respectively, while the R^2^ for RF and SVM were 0.85478 and 0.66591 respectively. This means that 90.69% of the predicted travel times correlated with the actual travel times when the XGBoost algorithm was applied to the blind data set (data from well C), while 85.49% and 66.59% of the predicted travel times correlated with the actual travel times when the RF and SVM algorithms were respectively applied to the blind data set.

The XGBoost also exhibited the lowest errors as compared to the RF and the SVM algorithms, as evidenced by the MAE, MSE, and RMSE values in Table 6, thereby making it the most accurate in its predictions during the blind set validation. The RF algorithm was also more accurate than SVM, having a higher R^2^ and lower MAE, MSE, and RMSE values than the latter.

Fig. 12 compares the predicted sonic logs (red line) and original sonic logs (blue line) following the application of the three algorithms on the blind data set. A strong correlation is also observed between the predicted and original sonic logs, as supported by Table 6 below.

The sonic or acoustic log measures the travel time of an elastic wave through a formation. Therefore, from Fig. 12, both the predicted and actual travel times of the elastic wave in well C decrease with increasing depth except in a few areas where a slight increment in the travel times was observed at deeper depths. This implies that the velocity of the wave also varies with increasing depth. It increases with decreasing time and decreases with increasing time because of the inverse relationship between velocity and time.

The speed or velocity with which waves travel through formations depends on a number of factors, including lithology, rock textures, porosity, etc. Notably, velocity decreases with increasing porosity and increases with decreasing porosity or increasing density. The varying wave velocities observed in well C may, therefore, be attributed to variations in the composition or characteristics of the subsurface layers. The high-velocity zones may be highly dense or compact zones and the low-velocity zones may be characterized by some porosities.

The behavior of the gamma ray, resistivity, neutron-porosity, and density logs in the same well (Fig. 4 above) suggest that the formation is indeed characterized by different porosities and lithologies, presumably sandstone, limestone, and shale. Between 5000 ft and 6000 ft, the formation's radioactivity is relatively low (about 0–50 API) with correspondingly lower resistivity and density and relatively higher porosity and travel time (lower velocity). This suggests that lithology in this zone could be slightly porous sandstone. The area between 6000 ft and 8000 ft may be characterized by sandstone and limestone interspersed with shale given the slightly higher radioactivity of the formation, relatively higher resistivity and density, and reduced porosity and interval transit time (higher velocity). From 8000 ft to 12,000 ft, there is a slight drop in resistivity, a marginal increase in porosity, a marginal drop in density around 9000 ft, a slight increase in travel time, and a slight increase in the formation's radioactivity. This area may be dominated by sandy-shale and shale lithologies with small amounts of porosity.

However, interpretation of the logs is not the scope of this work as additional information would be required to confirm the exact characteristics of the subsurface. The interpretation above is to emphasise that synthetic logs can provide the true reflection of the subsurface given the strong correlations observed between the predicted and actual sonic logs and the rest of the logs in the well.

The estimated velocities from the sonic log (whether synthetic or measured) aids in the determination of reservoir properties such as porosity, permeability, lithologies, elastic parameters, etc., which are critical for oil and gas exploration and production. The strong correlation observed in Fig. 12 between the actual and predicted compressional sonic log in well C affirms that logs that are missing in logging suites can be predicted based on available log data and can be used for formation evaluation.

Given the degree of correlation between the predicted and original sonic logs in all three cases, it suffices to conclude that all three algorithms can accurately predict or synthesize sonic logs if the sonic log is missing in a logging suite. However, the ensemble algorithms may be prioritized in such predictions because of their superior prediction capabilities.

The results have demonstrated that the ensemble methods (XGBoost and RF) are more accurate than the non-ensemble method (SVM) which agrees with the studies reported by Ref. [36] and others.

From Table 4, Table 5, Table 6, RF exhibited the best performance during training and testing but underperformed relative to XGBoost when it was deployed on the blind data set (well C). Since XGBoost builds the model sequentially, identifying incorrect predictions and improving on their prediction accuracies until a satisfactory model is obtained, this algorithm probably had a better understanding of the patterns in the data than RF and hence exhibited superior generalisation ability in this study.

From Fig. 12, all three algorithms exhibited high prediction errors at depths between 5000 and 5300 ft and around 10,050 ft, and SVM alone had high errors from 10,050 ft–12000 ft. It is possible that the characteristics of the data or the subsurface in these locations are different from the subsurface characteristics at similar depths in wells A and B, thereby accounting for these errors and the behavior exhibited by the algorithms.

## 6 Conclusion

This study employed SVM, RF, and XGBoost ML algorithms to predict the compressional sonic log from commonly acquired logs (gamma ray, neutron porosity, density, and resistivity) from wells in the Western (Tano) sedimentary of Ghana (West Africa). The study also ascertained the veracity of claims that ensemble ML algorithms are more accurate than non-ensemble ML algorithms by comparing the performance of the SVM (non-ensemble algorithm) with the performances of RF and XGBoost, which are ensemble algorithms. Data preprocessing, use of relevant input parameters, optimized hyperparameter selection, and cross-validation of supervised ML algorithms are essential steps to ensure confidence in the predictions made by ML algorithms. All these steps were employed in the prediction of the compressional sonic log in this study. The outcome of the study proves that the SVM, RF and XGBoost algorithms can successfully predict the compressional sonic log when it is missing in a logging suite. However, the ensemble algorithms (RF and XGBoost) should be prioritized when predicting the sonic log or any other log because of their superior generalization abilities to the non-ensemble method (SVM). This study agrees with previous works that reported that the ensemble algorithms are more accurate than the non-ensemble algorithms. Despite the superior performance of the XGBoost, the study cannot confirm that the XGBoost algorithm will always outperform the RF algorithm.

Generating synthetic logs has the potential to reduce greenhouse emissions from oil and gas exploration and production activities, as emissions resulting from the use of rigs to acquire logs will be avoided. ML could, therefore, be an essential tool, in this era of energy transition, for the oil and gas industry to cut down carbon dioxide emissions and continue to provide the global energy needs.

To the best of the knowledge of the authors, this is the first study that has employed machine learning algorithms to predict the compressional sonic log using well data from a Ghanaian sedimentary basin and is one of the few such studies in the whole West African sub-region. The study would, therefore, be very useful for hydrocarbon exploration and production in Ghana, the West African sub-region, and the world at large as it will help accelerate and enhance understanding of oil and gas fields and help save time and cost of sonic log acquisition. In subsequent studies, the authors will use these or other ML algorithms to predict sand and shale volumes from logs in the study area.

## Author contribution statement

Callistus Nero; Conceived and designed the experiments.

Performed the experiments, Analysed and interpreted the data, Contributed regents, materials, analysis tools or data, Wrote the paper.

Akwasi Acheampong Aning; Conceived and designed the experiments, Analysed and interpreted the data, Contributed regents, materials, analysis tools or data, Wrote the paper.

Sylvester Kojo Danuor; Conceived and designed the experiments, Analysed and interpreted the data, Contributed regents, materials, analysis tools or data, Wrote the paper.

Victor Mensah; Conceived and designed the experiments, Analysed and interpreted the data, Contributed regents, materials, analysis tools or data, Wrote the paper.

## Data availability statement

The authors do not have permission to share the data.

## Declaration of competing interest

The authors declare that they have no known competing financial interests or personal relationships that could have appeared to influence the work reported in this paper.

## Figures and Tables

**Table:** 

DT	Sonic log
GR	Gamma Ray log
MAE	Mean Absolute Error
ML	Machine Learning
MSE	Mean Squared Error
RES	Resistivity log
RF	Random Forest
RHOB	Density log
RMSE	Root Mean Squared Error
SVM	Support Vector Machines
XGBoost	Extreme Gradient Boosting
